# Supplementary material for: Are carbon emissions trading and green financial instruments synergistic? -Comprehensive quantitative research based on content analysis
Source: PLoS One. 2024 Mar 7;19(3):e0298601. doi: 10.1371/journal.pone.0298601 (PMC10919601; doi:10.1371/journal.pone.0298601)
Supplement: S1 Data — (ZIP) [file pone.0298601.s001.zip › Data/地级市Z_F工作报告.zip!/╕╩╦α/╛╞╚¬╩╨/└┤╫╘╣1⁄2╓┌║┼í╛┬φ┐╦╩2╛▌═°í┐_2009.docx]

各位代表：

　　现在，我代表酒泉市人民政府向大会作工作报告，请予审议，并请各位政协委员和列席人员提出意见。

　　2008年工作回顾

　　2008年，是国内外经济形势异常复杂、重大事件较多、应急任务较重的一年。一年来，我们在中共酒泉市委的正确领导下，以党的十七大精神为指针，以科学发展观为统揽，认真贯彻省委、省政府和市委的工作部署，落实全市工业大会、农业大会、城市规划研讨会精神，团结带领广大干部群众艰苦奋斗，扎实工作，较好地完成了市二届人大三次会议确定的任务目标，实现了全市经济社会又好又快发展。

　　--经济增长速度较快。预计全市生产总值240亿元，增长16%，为近年来增长最快的一年；完成固定资产投资157.2亿元，增长35.5％；实现社会消费品零售总额63亿元，增长22%。投资规模扩大，消费需求旺盛，增长势头强劲，成为全市经济发展的显著特征。

　　--经济效益明显提升。财政总收入突破20亿元大关，地方财政收入突破10亿元大关，分别达到21.7亿元和10.03亿元，增长26.1%和32%，明显高于经济增速。规模以上地方工业实现利税7.3亿元，增长46.9%，综合效益指数200.8%，提高54.7个百分点。

　　--生态治理取得实效。节水工作扎实推进，亩均耕地用水496立方米，比去年减少2%，湿地保护、风沙治理、造林绿化等生态工程取得新的进展，我市荣获"全国三北防护林体系建设突出贡献"荣誉称号；节能减排完成确定的约束性指标，万元生产总值能耗1.61吨标准煤，下降5.2%，主要污染物二氧化硫排放控制在2.1万吨，化学需氧量减排2016吨，下降2.65%。

　　--群众生活更加殷实。城镇居民人均可支配收入12621元，农牧民人均纯收入5761元，分别增加1352元和655元，增长12%和12.8%；居民消费结构升级，购房、购车、教育、通讯等消费支出增加，城乡居民恩格尔系数下降到32.2%和35.1%，人口自然增长率控制在3.19‰，居民生活质量进一步提高。

　　--民生问题得到改善。社会保障覆盖面扩大，启动实施离任村（社区）干部和"三老一少"生活补助制度，提高城乡医疗统筹标准和报销比例，制定"低保边缘户"救助政策，使广大群众特别是困难群众得到更多的实惠；就业面继续扩大，城镇新增就业1.08万人，登记失业率控制在3.65%；政府承诺的安全饮水、廉租房建设、街巷改造等惠民实事得到较好落实。

　　这些成就的取得，标志着我市经济实力进一步增强，发展方式进一步转变，社会和谐程度进一步提高，我们在科学发展的伟大实践中又迈出了坚实的一步。一年来的主要工作是：

　　一、狠抓项目建设，加快优势产业开发

　　坚持发展抓项目，把握政策机遇，抢抓市场机遇，加快项目建设和优势产业开发，增强地方经济实力。

　　项目建设成效显著。继续完善项目引进、包抓包挂、考核奖励机制，加大项目前期投入，优化项目投资结构，全市开工建设亿元以上项目60项，增加24项，千万元以上项目414项，增加77项，项目建设呈现出大项目多、资金到位好、建设进度快的特点。基础设施建设步伐加快，灌区续建配套、水库除险加固、农田水利工程完成建设任务；嘉瓜330千伏输变电工程建成，750千伏输变电工程完成"四通一平"；市域铁路网中长期规划通过省上论证，敦煌、玉门火车站全面建成，敦格铁路、淖柳铁路、酒航铁路前期工作进展顺利；瓜猩高速、敦当公路、酒嘉快速通道完成报批，新建、改建农村道路1002公里，建成14个乡镇汽车站和117个行政村停靠点。

　　工业发展势头强劲。强化工业主导地位，提出了以风电开发为牵引，煤电、网架、风机制造相配套，农产品加工、矿产品选炼、石油化工为支撑的发展思路，力争五至六年工业增加值翻两番。清洁能源基地建设加紧实施，380万千瓦风电场建设完成特许权和设备采购招标，8个风电项目开工建设，瓜州北大桥、大梁西二期风电项目建成发电，建成和在建风电装机容量121万千瓦。煤电建设和风机制造加快跟进，酒泉热电厂完成"五通一平"和工程、设备招标，常乐电厂、明沙窝电厂完成可研审查。酒泉风光电装备制造产业园列为省级基地，签约建设项目14个，签约投资32亿元，中航惠腾风轮叶片和金凤、华锐科技风机总装等5个项目开工建设。新建、续建水电站14座，有7座建成发电，新增水电装机3.5万千瓦，累计装机21万千瓦。核乏料后处理项目完成厂址勘察和初可研报告，敦煌10兆瓦光伏发电项目列入国家规划，已开始特许权招标。加快矿产资源开发，46个矿产勘查项目列入省上出让计划，新建、扩建矿产品加工项目116个，铁、钒、铅锌、原煤生产能力扩大，成为地方工业发展的重要支柱。工业园区聚集功能增强，酒泉中小企业创业园建成，玉门建化工业园列入全省循环经济试点。骨干企业成长壮大，销售收入过亿元的工业企业达到18户，新增10户，过千万元的达到165户，新增60户。全市完成工业增加值100亿元，增长17%。

　　"一特四化"全面启动。按照服务引领农户、培育竞争实力的思路，制定了"一特四化"实施意见，提出了发展节水高效特色农业的任务目标。落实支农惠农政策，争取农业专项资金1.5亿元，发放粮食、农资、农机具、奶牛良种、能繁母猪等涉农补贴资金1.02亿元，农民人均受益175元。扩大日光温室、网室制种、优质林果、啤酒原料等产业规模，新增高效特色产业面积7.2万亩，是近年来发展最快的一年。启动实施万户规模养殖工程，建成标准化养殖小区651个，规模化养殖场户2.73万户，规模饲养量占全市畜禽饲养量的62.6%。提高产业化经营水平，建立农村专业合作经济组织214个，建成农产品加工流通企业459户，当年新续扩建24户，农产品加工能力达到150万吨，带动基地165万亩，占农作物播种面积的80%。组建农产品营销协会，举办特色农产品节会，开通大宗农产品运销专列，加大农产品促销力度，全市60%的农产品实现外销。制定种植业、养殖业技术标准，开展无公害产地、产品认证，落实动植物疫病防控责任制，狠抓苹果蠧蛾防控和农畜产品质量监管，确保农业生产和食品卫生安全。多层次开展技能化培训，输转农村劳动力11.46万人，劳务收入占农民人均纯收入的25%。九甸峡移民安置基本完成，安置移民1948户9357人，全市移民人均纯收入1654元，增长5.8%。扎实推进新农村建设，狠抓村组道路硬化、自然村整体改造和村容村貌整治，新建"一池三改"沼气用户7026户，累计达到17820户，新建和改造农村小康住房13886套。

　　改革开放继续深化。完善敦煌种业公司治理结构，探索建立股权激励机制。理顺电力、烟草管理体制，组建成立酒泉供电公司、超高压输变电公司和烟草公司。市公路工程局完成破产重组，金塔、瓜州、敦煌农村公路养护改革列入全省试点。资源市场配置化程度进一步提高，出让土地1896亩，招拍挂处置占92%。扩大国库集中支付范围，完善非税收入管理，强化财政支出重点项目绩效考评。制定市直院校、医院引进人才激励政策，拓宽引智引才渠道。健全地方金融服务网络，兰州银行酒泉分行、酒泉邮政储蓄银行、敦煌农村合作银行和金盛村镇银行挂牌运营，兴昌投资、新广源信用担保公司成立。对外开放领域不断扩大，组团参加科博会、浙洽会、乡洽会等重点节会，举办第六届银企合作项目推介会，落实签约资金29.16亿元，瓜州、玉门、敦煌等县市举办的投资峰会、产品展销会成效明显。全市共签约招商引资项目186个，开工建设184个，实际到位资金53亿元，增长69%。外贸进出口企业达到84户，实现进出口总额5000万美元，增长13.6%。

　　二、提升城市品位，促进商贸旅游发展

　　完善城市基础设施，提高综合承载能力，发挥城市聚集辐射功能，活跃商气，聚集人气，促进第三产业快速发展。

　　城市建设步伐加快。全市完成城市建设投资18.7亿元，增长56%，是近年来投资最多的一年，有力地推动了城市建设。高起点规划城市，邀请知名专家评审修编城市规划，启动编制市域城镇体系规划和历史文化名城保护规划，完善酒泉老城区控制性详规，完成51个乡镇、160个村庄的村镇规划编制。建设精品城市，加快重点市政工程建设，稳步推进自强市场、南关小学等片区拆迁，建成世纪大道北段、阳关路西段等12条道路，完成49条街巷和东关楼群道路改造，临街主要建筑物美化亮化工程基本完成。狠抓市政道路、园林和城市出入口绿化，新增公共绿地96.5万平方米。新建高标准公交站亭51座，更新出租车314辆。精细管理城市，启动供热分户计量试点，制定城市道路、供热、公厕管理办法，深入开展"迎奥运、爱酒泉"环境卫生综合整治活动，省级文明城市、卫生城市和园林城市创建通过省上验收。县市城区面貌明显改观，敦煌市城市道路及夜景灯光改造、金塔县"三湖一溪"建设及集中供热改造、玉门新市区绿化及昌盛路建设、瓜州县老城区街道改造、肃北县外环路建设、阿克塞县水上乐园等亮点工程为城市再添新景。

　　商贸流通繁荣活跃。加快区域性商贸中心建设，世纪新天地商业广场、西部奇石市场、敦煌风情城建成运营，富康购物中心灾后重建开业。深入实施便民服务市场工程，推进名优商品、营销网点进乡村、进社区，新建改建"农家店"200个，累计达到777个，覆盖农业县市90%的乡镇和70%的行政村，敦煌市列为全国农村商务信息化试点。引进新型流通业态，北京华联、兰州西太华入驻酒泉，西部、西洞信息物流园建成运营，新增商业品牌店、直销店、加盟店65个，全市累计建成商业批发、零售网点1.2万个，农资配送中心17个，营业面积210万平方米，我市已成为河西地区规模最大的商流物流中心，人均消费品零售总额居全省第三。举办首届酒泉美食节，成立饮食服务业协会，制定行业自律规则，对5434个餐饮和服务企业进行等级评定。加强食品、药品、农资、屠宰、废旧物品回收等专业市场监管，与省上签订的产品质量、食品安全责任书任务全面完成，市场秩序进一步好转。

　　旅游环境不断优化。深入实施"双十双百"旅游发展计划，新建、续建重点旅游项目34个，酒泉文化旅游产业园加快建设，敦煌党河风情线完成水体工程，酒泉博物馆完成装修布展，铁人纪念馆建成开放，梦柯冰川接待中心主体工程竣工，敦煌、瓜州、阿克塞博物馆加紧建设。完善旅游配套设施，敦煌沙州大酒店投入运营，新增A级景区3处、星级饭店6家、农家乐30家，新建星级旅游厕所12座。提高旅游服务水平，酒泉宾馆被评为"中国十佳旅游度假酒店"，11家旅游饭店和旅游景区晋星升级，3家旅行社进入全省五强，我市荣获"中国旅游品牌魅力城市"称号，敦煌市被评为"中国最佳生态旅游目的地"城市。以神七发射、奥运火炬传递为契机，加大旅游宣传促销，组团参加国内外旅游推介会，举办敦煌飞天文化旅游节和梦柯冰川攀登活动，增加敦煌旅游航班和专列，全市接待海内外游客305万人次，增长13.7%，完成旅游收入18.5亿元，增长12.8%。

　　三、建设生态文明，增强可持续发展能力

　　认真贯彻中央、省委对生态保护的重要指示，狠抓节水改造、生态治理、节能减排和资源综合利用，加快生态文明建设。

　　节水措施进一步强化。以农业节水为重点，完成农业用水初始水权分配，全面推行定额管理、超定额累进加价和阶梯式水价制度，采取关井、限采、提价、压田等强制性措施，减少农业灌溉用水，累计关闭机井和自备水源井286眼，安装智能水表3519块，占农用机井的41%，压减灌溉面积3.41万亩。继续实施百万亩节水灌溉工程，全市高效节水面积达到10.5万亩，新增2.25万亩，其中推广河水滴灌3016亩。制定和完善高效节水工程实施方案，8个灌区续建配套与节水改造项目完成年度建设任务，月牙泉水位下降应急治理工程投入使用，水位比2007年上升132厘米。

　　生态建设取得实效。高度重视戈壁和自然保护区管护工作，严格禁止在湿地放牧、开荒、开矿，实施草原"三化"治理、退牧还草、封滩育林等生态工程，完成人工造林7.2万亩，建设绿色通道319公里，完成防风治沙3.8万亩。敦煌生态环境和文化遗产保护项目上报国务院审批，瓜州生态系统研究试验项目和中蒙边境草原防火隔离带工程启动实施。

　　"双控"目标较好落实。强化节能降耗和污染物减排目标责任制，建立新上项目能耗评估审查制度。狠抓集中供热、建筑节能、市政照明、城市公交和出租车节能技术改造，淘汰落后产能43.1万吨，对年耗能2000吨标准煤以上的49户企业和8户重点排污企业实施动态监控，取缔"两高一低"企业15户。实施节能减排项目26个，争取扶持资金4350万元。西脉公司废气制酸项目建成投产，酒泉城区沙尘暴监测站投入使用，酒泉污水处理厂升级改造工程通过审批立项，即将开工建设，玉门、敦煌、青海油田生活基地污水处理厂建成运营。

　　资源保护力度加大。坚守耕地保护红线，全市耕地总量达到230.2万亩，基本农田保护率达到85.6%。矿产资源规划修编和整合工作基本完成，加快整合石棉、铁矿等矿山企业，全市18户煤矿、12户金矿分别整合为6户，关闭小煤矿5家。严格执行矿山开采与生态保护"三同时"规定，实行矿山环境恢复保证金制度，申报地质遗迹保护、环境治理恢复、矿产资源保护等重点项目14个，有效整治无证开采、破坏环境、浪费资源等突出问题。

　　四、着力改善民生，保持社会和谐稳定

　　把关注民生、维护民利作为政府工作的重中之重，认真落实各项惠民政策，全面发展社会事业，促进社会和谐进步。

　　就业渠道不断拓宽。落实就业再就业扶持政策，财政支出就业补助资金4195万元，增加480万元，帮助4818名下岗失业人员实现再就业。健全就业援助制度，政府购买公益性就业岗位1123个，安置零就业家庭就业181人，选派532名高校毕业生到基层就业。广泛开展创业促就业行动，为427名下岗失业人员发放小额担保贷款1767.2万元，带动就业3000多人。加强劳动保障执法监察，推行建设领域农民工工资保证金制度，清欠工资630万元，农民工劳动合同签订率达到86.1%。

　　民生得到较大改善。完善城镇职工基本养老、医疗、失业、工伤保险制度，提高城乡低保补助标准，为城乡13.37万名低保对象发放低保金和物价补贴9811万元，增加3969万元，6.49万名贫困移民和7713名残疾人纳入城乡低保范围。基本医疗统筹、大病统筹、城乡医疗补助及报销标准全面提高，城镇居民基本医疗参保面达到90.1%，农村新型合作医疗参合率达到94.67%，为城乡困难群众发放医疗救助金772.7万元。建立"三老一少"生活补贴制度，高龄老人、困难老人、残疾老人生活补助标准大幅度提高，特困家庭大学生每学年补助生活费1000元。住房公积金制度覆盖面扩大，低收入职工购房能力增强。制定廉租房建设五年规划，新建廉租房1165套，发放租房补贴9090户，实物配租1253户，廉租住房保障工作全省考核第一。农村安全饮水工程超额完成年度任务，又解决了5.4万名农村居民安全饮水问题，累计解决16.8万人。积极发展扶老、助残、济困等社会福利和慈善事业，改造贫困群众和残疾人危房365户，资助182名残疾学生就学，县市区福利院、敬老院、五保家园建成或开工建设。广泛开展民族团结进步项目对口帮扶活动，为瓜州县腰站子东乡族乡捐助资金650万元；动员干部群众为地震灾区献爱心，捐款1697万元。

　　社会事业全面发展。财政用于科技、教育、文化、卫生、体育等社会事业支出6.4亿元，增长39.7%。以项目为载体，推广应用新技术188项，16项科技成果通过省上鉴定，47项科技专项得到国家和省上支持，大禹公司研制的滴灌管项目列入国家"948"科技计划，并与国家水利科学院进行股份合作。加快农村教育布局调整，对办学规模较小的75所乡镇中小学进行撤并，改建九年一贯制学校2所，新建和扩建寄宿制初中5所，肃北、阿克塞高中教育在敦煌办学，全市农村高中生和25个乡镇的初中生实现进城就读。酒泉市新城区新苑学校建成，新增办学规模2100名。争取中央补助资金，化解农村中小学遗留债务1.26亿元。"两免一补"政策实现城乡全覆盖，农村中、小学公用经费分别提高50%和33%，县镇中、小学分别提高39%和21%，贫困家庭初中、小学寄宿生生活补助分别提高214%和109%，免费教科书发放范围扩大到农村、县镇所有学生和城市贫困家庭学生，发放面达到81.5%。积极应对突发公共卫生事件，及时处置疫情传播，免费对2.03万名食用"问题奶粉"的婴幼儿进行检查治疗，公立医院设置济困病床229张，"仁和家园"小区惠民医院建成。文艺创作水平提高，广场、节庆文化和文化"四进"活动丰富多彩，酒泉图书馆、敦煌大剧院建成使用，酒泉飞天数字影院、敦煌动漫网络制作中心开工建设，建成综合文化站7个、"农家书屋"215个，6个文化馆被评定为国家级文化馆。农村广播电视无线覆盖工程通过省上验收，数字电视传输至酒泉卫星发射中心。认真落实计划生育扶持政策，低生育水平进一步巩固。精心组织奥运火炬传递活动，举办全市第二届运动会和第六届民族运动会，开展日全食科普观测活动，酒泉体育场竣工，新建农民健身广场10个，"一村一场"建成率达到40%以上。《酒泉市志》出版发行，人防指挥自动化系统进一步完善，防震减灾工作获全国一等奖，我市被命名为全国双拥模范城。

　　平安建设稳步推进。深入开展"平安酒泉"创建活动，落实安全生产问责制，对道路交通、矿山企业、危险化学品等重点领域和火灾隐患进行排查治理，安全隐患整改率达到86.5%，安全生产四项控制指标全面下降。组织开展矛盾纠纷排查化解、重信重访专项治理和"一把手"大接访活动，一批信访积案和上访案件得到妥善处理，群众来信来访、集体上访和非正常越级上访大幅下降。加强人民调解工作，开展"法律六进"活动，"五五"普法中期考核评估获全省先进集体。全力抓好迎奥运、神七发射等重大活动安全保卫工作，推进"三队、三所"建设，完成"三基"建设项目231个，在全省率先建成警务综合信息平台和办公自动化系统。加强社会治安综合治理，严厉打击盗窃、抢劫、贩毒等各类犯罪活动，群众安全感进一步增强。

　　五、加强自身建设，提高政府行政效能

　　按照为民、务实、清廉的要求，加强政府自身建设，开设"行风面对面"电视专栏，30个行政执法部门和窗口服务单位公开接受群众评议。征求省直部门对市直单位的评价意见，公布评价结果，督促市直机关提高工作效能。扩大政务公开范围，编制行政职权目录及工作流程，建成市政府采购服务大厅，建立市政府信息公开网站，增强政府工作透明度。加强政务大厅管理，快捷办理审批事项，受理办件33万件，办结率达99.8%。加快电子政务建设，政务专网和办公自动化系统建成运行。增强财政公共服务职能，积极争取财政转移支付资金，保证重点支出及时到位，完成财政支出38亿元。落实依法行政责任制，对56个具有执法职能的部门进行考评。完善民主决策程序，对涉及群众切身利益的收费事项进行公开听证，对城市规划、风电煤电建设、"一特四化"等重大方案组织专家评审论证。自觉接受人大依法监督和政协民主监督，认真办理人大代表建议和政协提案。统计监测服务水平提高，价格监管和市场化改革双向推进。加强财政预算执行审计、民生资金审计、政府投资项目审计和领导干部经济责任审计，行政监察和审计监督卓有成效。

　　各位代表，2008年是我们克服困难、应对挑战，经济社会发展取得明显成效的一年。这些成绩的取得，是我们深入学习实践科学发展观，贯彻省委、省政府正确决策的结果，是市委坚强领导和市人大、市政协大力支持的结果，是全市人民奋力拼搏、辛勤努力的结果。在此，我代表市政府，向人大代表、政协委员和广大干部群众，向各民主党派、无党派、工商联、人民团体、离退休老同志、驻酒人民解放军、武警官兵、公安干警和中央、省属驻酒单位以及所有关心、支持酒泉发展的各界人士，致以崇高的敬意和衷心的感谢！

　　在总结成绩的同时，我们也清醒地认识到，政府的工作还存在着一定的差距和薄弱环节，主要是驾驭市场经济的能力还不强，应对金融危机、保持平稳发展的思路和办法还不多；税源结构单一，财政收入主要依赖资源开发，市级财政收支压力较大，许多想办、快办的惠民实事难以付诸实施；农民增收的难度或制约因素增多，移民群众生产生活较为困难，扶贫开发任务繁重；改革发展中的矛盾纠纷依然较多，刑事发案上涨较快，不稳定因素不容忽视；政府工作效能还不适应加快发展的要求和群众的愿望，机关作风还有待进一步改进。这些问题，我们要高度重视，努力在今后的工作中加以改进和解决。

　　2009年工作思路和目标

　　今年，我们面临着严峻的形势和繁重的发展任务。全球金融危机愈演愈烈，迅速向实体经济扩散，向内陆地区传导，外部市场萎缩给我市产品输出带来较大影响，企业经营困难增多，经济下行压力加大，就业形势比较严峻。尽管我们面临着诸多困难和复杂多变的外部环境，但我市经济发展的优势依然存在，重要战略机遇期依然存在，长期增长的趋势没有改变。国家及时调整宏观政策，强力启动内需，给我们加快发展带来了新的契机。我市地域辽阔，基础设施投资有较大的空间；生态环境脆弱，生态治理倍受国家和省上关注；农业比较优势明显，农业投入有较好的前景；自然资源富集，承接东部产业西移有较好的条件；消费水平较高，服务业发展有较大的潜力；多年来我们坚持不懈抓项目，一大批符合国家产业导向的项目已完成规划和正在加紧规划。这些都为我们争取政策投资、扩大招商引资、加快经济发展带来了难得的机遇。

　　今年政府工作的总体要求是：深入贯彻党的十七大、十七届三中全会精神，以科学发展观为指导，认真落实中央宏观调控政策，按照省委、省政府"四抓三支撑"的战略部署，强化工业支撑，夯实农业基础，促进农民增收，推进城乡一体化进程，努力实现经济社会又好又快发展。

　　统筹考虑诸多有利因素和不利因素，2009年全市经济社会发展的预期目标是：地区生产总值270亿元，增长12%，财政收入24.3亿元，增长12%，其中地方财政收入11.4亿元，增长13.5%。固定资产投资突破200亿元，增长30%，社会消费品零售总额76亿元，增长20%，城镇居民人均可支配收入增长10%，农牧民人均纯收入增长12%。居民消费价格涨幅控制在4%以内，万元生产总值能耗降低5%，主要污染物排放减少2%，人口自然增长率控制在6.5‰以内。实现上述目标必须把握好五个重点：

　　坚持项目带动，把扩大投资作为快速发展的支撑。投资对生产和消费具有双重拉动作用，是推动我市经济增长的主要变量。去年年底和今明两年，国家将安排4万亿元投资用于基础设施建设、生态环境保护、社会事业发展、改善民生和涉农服务。我们要抓住这一政策机遇，认真谋划对接项目，继续实施领导包抓、部门齐抓、协作攻关、考核奖励等工作机制，争取更多的项目、更多的投资入驻我市。坚定不移地推进"一特四化"进程，加快风电及配套产业开发，实现工农业发展新突破。抓住东部地区产能过剩、产业梯度西移的有利时机，引进知名企业，引进适用技术，改造提升传统产业，抢占经济复苏先机，培育新的经济增长点。

　　坚持消费拉动，把扩大内需作为平稳发展的基础。平稳发展是科学发展的基本要求，实现平稳发展，防止大起大落，必须扩大消费，增强消费对经济增长的拉动作用。充分发挥我市中央省属单位较多、地处四省区交汇要道、聚集辐射优势明显的区位条件，加快商贸城建设，引进知名批发商，培育民间经销商，形成大批量输入、大批量输出的集散格局。努力诱发市内需求，扩大周边需求，确保消费持续升温，服务业繁荣活跃，房地产平稳发展，旅游业快速增长，为第三产业发展创造良好环境，努力扩大就业规模。

　　坚持执政为民，把改善民生作为和谐发展的根本。牢固树立公仆意识，时刻情系群众，多想惠民之策，尽心为民谋利；深入调查研究，主动问政于民，问需于民，问计于民，使政府的决策更合民意，工作更有效率；结合工作实际，广泛开展承办惠民实事活动，着力解决群众的急事、难事、怨事，将服务职能向社会延伸，工作重点向基层下沉，切实将关注民生付诸于工作实践。加强党风廉政建设，加大反腐倡廉力度，坚决纠正损害群众利益的不正之风，以政风建设带动和谐社会建设。

　　坚持生态立市，把节约用水作为永续发展的关键。广泛开展基本市情教育和可持续发展教育，增强危机意识，将保护资源、节约用水变为全民的自觉行动；认真研究市场化节水的新举措，逐步提高水价，引导节水投入，提高节水效率；按照节水与增效的双重目标，大力调整种植结构，扩大高效节水面积，通过增效筹集节水资金，通过节水实现长远增效；加大行政执法力度，贯彻禁荒、禁井、禁移"三禁"决定，积极探索多种节水模式，推进水务一体化建设，遏制地下水过度开采，促进生态修复。

　　坚持依法行政，把提高效能作为政风建设的核心。把依法办事作为政府工作的基本准则，把提高效能作为政府工作的不懈追求，推进政府自身建设。认真履行依法行政责任制，严格依法办事，规范从政行为，把住从政"底线"。加强民主政治建设，更广泛地扩大民主范围，更全面地听取各方意见，更规范地履行民主程序，使各项决策最大限度地赢得群众的支持和拥护。树立行政成本意识，节约民财，珍惜民力，减少务虚开支，反对形式主义和铺张浪费。继续深化审批制度改革，快捷办理审批事项，提高工作效率，树立政府良好形象。

　　2009年主要任务

　　保持全市经济社会平稳较快发展，实现年度工作目标，我们将重点抓好十个方面的工作：

　　1、全力做好项目工作，努力扩大投资规模。抓住国家扩大内需的有利时机，快选项目，多选项目，选好项目，提高项目建设水平，确保固定资产投资较快增长。在水利建设上，继续抓好水库加固、灌区改造、河道治理等水利工程，编制敦煌水资源合理利用与生态保护综合规划，做好引哈济党前期工作。在交通建设上，开工建设瓜猩高速、敦当公路、酒嘉快速通道和酒航公路至火车站、敦煌至七里镇公路，加快酒泉、敦煌国家级枢纽客运站建设，争取敦格铁路和兰新铁路客运二线酒泉段开工建设，完成酒航铁路报批立项。在能源建设上，力争建成酒泉热电厂主体工程，开工建设750千伏输变电工程、小宛30万千瓦国产化示范风电场、柳园5万千瓦风电场、明沙窝电厂和敦煌10兆瓦光伏并网发电项目，做好常乐电厂、昌马电厂、红柳洼电厂等项目前期工作。抓好项目招投标、建设进度、资金监管等工作，提高工程质量和投资效益。

　　2、加快"一特四化"进程，大力发展现代农业。以专业化布局带动特色产业发展。按照稳粮压棉增特色的思路，加快种植结构调整，制定六大产业布局规划，扩大日光温室、鲜食葡萄、网室制种、啤酒花、果蔬等特色产业面积，力争万元田达到10万亩。继续实施规模化养殖工程，加快品种改良，新增肉牛2万头、肉羊40万只。以产业化经营提升农业市场竞争力。加快建设种子精选、酒花加工、肉品保鲜等产业化龙头项目，建立龙头企业与农户之间的利益共享机制，扶持发展农民专业合作社，提高农民进入市场的组织化程度。以标准化生产打造地域特色品牌。大力推行农产品技术标准、质量标准和卫生安全标准，编印重点产业标准化技术手册，建立设施农业和大田蔬菜标准化生产基地40万亩、标准化生产示范区30个，认定、认证无公害农产品产地和产品各10个。以技能化培训促进农民增收。整合培训资源，抓好种养技术和务工技能培训，输转劳务10万人。大力推进新农村建设，继续抓好"一池三改"和村组、居民点道路硬化，新建沼气池1.28万个，新建改造农村抗震安居住房1万套。

　　3、培育壮大优势产业，扶持发展中小企业。深入贯彻全市工业大会精神，加强工业发展前瞻性研究，化解金融危机影响，着力保增长、增效益，力争全市工业增加值达到120亿元，增长20%以上。完善园区基础设施，优化工业布局结构，制定出台五大产业集中布局、工业企业跨区域入园的激励政策。加快建设清洁能源基地，抓好风电、煤电、水电开发及网架、风机制造等配套项目建设，建成风机制造项目7个，力争风电、水电装机容量达到150万千瓦和24.5万千瓦。实施资源转换战略，大力发展以矿产品冶炼为重点的高载能产业，提高铁、钒、铅锌、原煤等优势资源加工水平，加快开发硅、磷、镍、石材等矿产资源。认真落实全省发展循环经济实施意见，重点实施锅炉节能、尾矿利用等产业扶持项目。扶持中小企业发展，落实税费减免优惠政策，财政拟安排资金1800万元，用于中小企业贴息和项目前期费用，帮助企业克服困难，促进地方工业平稳较快增长。

　　4、发挥城市辐射功能，统筹城乡协调发展。完善城市基础设施和综合服务功能，提高城市综合承载力，满足群众多样化需求。发挥城市规划专家咨询委员会作用，加快编制市域城镇体系规划、历史文化名城保护规划和老城区改造规划，按照高起点规划、分阶段建设、特色鲜明、经济适用的原则，完成14个乡和59个村的规划编制。加快酒泉老城区改造和新城区配套建设，完成金泉路等5条道路建设，抓好园林绿化和城市出入口路段的亮化美化工程，新增城市绿地81.5万平方米。加快实施老城区防洪排涝工程和天然气入市工程，建设老城区热源厂，供热分户计量改造1.5万平方米。继续抓好县市城区道路、管网建设和集中供热改造，加快污水、垃圾处理项目建设进度，争取早日运营。积极探索城市管理市场化运作机制，上划规划权，下放管理权，推进城市管理向社区延伸、向楼院覆盖。坚持分类指导，统筹城乡发展，探索和制定土地流转、产业发展、公共服务等方面的统筹政策，加快"城中村"改造和撤村建居步伐，促进农村人口向城市转移，公共服务向农村覆盖，新增城市人口1万人，城市化率达到57.8%。

　　5、活跃商贸旅游市场，保持消费持续增长。继续抓好区域性商贸中心建设，加快解放桥、春光、昌兴等专业市场改造，开工建设巨龙商厦、南方装饰城二期工程。落实"家电下乡"补贴政策，实施便民服务市场工程，发展农家店连锁超市和加盟店100个，办好酒泉洋葱节、酒花节、美食节等节会，积极发展会展经济。扶持发展现代服务业，引导消费升级，扩大即期消费，促进消费持续增长。加快旅游设施建设，开工建设雅丹地质公园环形路、锁阳城景区、敦煌悦榕山庄等旅游项目，完成敦煌党河风情线景观工程，建成开放酒泉、敦煌、阿克塞博物馆。开发培育旅游热点，积极发展休闲度假和乡村旅游。扩大旅游宣传促销，拓展海外客源市场，力争接待游客340万人次，实现旅游收入23亿元，增长24.3%。

　　6、深入推进各项改革，提高对外开放水平。以推动科学发展、增强发展活力为目的，继续深化改革开放。全面实施事业单位岗位设置管理工作，做到因事设岗，因岗择人，人随岗走，以岗定薪，岗变薪变。建立以县为主、建管分离、政事分开的农村公路管养体制。深化财税管理体制改革，推行惠农资金"一册明、一折统"管理办法，做好增值税转型和燃油税开征工作。推进资源产品价格改革，建立生产要素和资源价格形成机制。稳步推进集体林权制度改革，完善农村土地征用和农民补偿安置制度，探索建立土地银行、转包、出租、股份合作等土地流转方式，促进农村土地流转。规范中小企业信用担保体系，建立政府主导的信用担保机构，完善"借、用、还"管理机制，努力增加信贷投放。扩大对外开放，提高引资质量，力争引进到位资金50亿元，进出口总额5500万美元，增长10%。

　　7、加大生态治理力度，推进发展方式转变。发挥市场杠杆作用，完善水权制度、水价制度和用水管理制度，开展节水示范单位创建活动。量化用水计量标准，再安装智能水表3000块，安装率由41%提高到70%。启动实施敦煌水资源合理利用和生态保护综合规划，重点抓好40万亩高效节水工程。广泛开展绿化造林，完成人工造林6.5万亩，实施封滩育林50万亩，全面封禁生态公益林，争取瓜州、肃北、阿克塞列入国家第二批退牧还草项目计划。转变发展方式，推广资源节约、替代和循环利用技术，开展循环经济和清洁生产试点，加强重点耗能和排污企业监控，抓好集中供热、余热利用等节能改造，淘汰落后产能，推广安装高效节能灯1万只，加快实施酒泉污水厂升级改造工程，完成节能减排任务目标。

　　8、全面发展社会事业，增强公共服务功能。加大公共服务投入，财政拟安排科技、教育、文化、卫生、体育等社会事业支出9.2亿元，增长25%。发挥科技项目的辐射带动作用，推广实用新技术100项，提升产业科技含量。继续整合教育资源，加快寄宿制学校建设，完成18个乡镇中小学布局调整，基本实现高、初中分离办学和农村初中学生进城就读。创新职业教育模式，抓好酒泉职业技术学院示范性高职院校建设项目，开工建设残疾人特殊教育学校。加强学校周边网吧管理，净化教育教学环境。完善城乡公共卫生服务体系，高度重视疫病防控，抓好市医院门诊大楼和农村卫生院所建设，提高公立医院床位补贴标准，落实乡镇卫生院在编人员工资财政供给政策，推行乡镇卫生院药品"零差价"。加快公共文化体系、文化市场体系和文化产业建设，重点实施农家书屋、基础设施、"非遗"保护、信息共享等四大工程，繁荣文艺创作，抓好"丝路"申遗、文物普查和文化遗产保护。扩大数字电视覆盖范围，完成广播电视"村村通"年度任务，建成移动多媒体广播电视。广泛开展全民健身活动，开工建设市体育馆和游泳馆。落实计划生育利益导向"三项制度"，抓好移民和流动人口计生管理，稳定低生育水平。深化公民道德教育和未成年人思想教育，开展双拥创建和军民共建活动，维护民族团结与宗教和顺，做好统计、供销、市志、档案、外事、人防、边防、残疾人及防震减灾工作。

　　9、关注解决民生问题，促进社会和谐进步。落实就业再就业扶持政策，多层次开展技能培训，多渠道开发就业岗位，做好高校毕业生、下岗职工、返乡农民工就业再就业工作，新增城镇就业7000人，城镇登记失业率控制在4%以内。扩大城乡社会保障覆盖面，建立就业与失业保险和最低生活保障联动工作机制，重点做好私营企业从业人员、城镇灵活就业人员和农民工参加社会保险工作，开展被征地农民保险试点，推行村干部养老保险制度。制定和完善城镇职工基本医疗保险政策，实施公务员医疗补助制度。继续提高企业退休人员养老金水平和优抚对象生活费补助标准，完善"三老一少"生活补贴制度和"低保边缘户"救助制度。切实做好安全隐患预警排查工作，严防重特大事故发生。加强民生物资和食品、药品、饮食卫生监管，确保人民群众身体健康。靠实领导信访责任，畅通群众诉求渠道，做好社会矛盾纠纷化解调处工作。积极开展法律援助，保障弱势群体合法权益。加快中心敬老院、社会福利院和救助管理站建设。健全公共应急管理体系，推进公安信息化、城市视频监控和农村技防建设，打击违法犯罪活动，维护社会安全稳定。

　　切实解决民生问题，办好十件惠民实事：一是购买公益性就业岗位800个，帮助零就业家庭、就业困难人员和下岗失业人员就业；二是实施"151创业促就业"计划，新增担保基金1000万元，为创业人员发放贷款5000万元，带动就业1万人；三是在酒泉市区新建寄宿制初中2所，增加办学规模7000人，力争新学年招生；四是为全市寄宿制学校修建公共浴室，年内50%的学校完成修建任务；五是财政列支专项资金，扶持发展高效特色产业10万亩，调引良种母羊3万只，培训农村劳动力10万人次，促进农民增收；六是新建改造农村道路1000公里；七是继续实施农村安全饮水工程，再解决5万名农村居民的安全饮水问题；八是新建廉租住房1000套、经济适用住房1000套，解决保障性住房1万户，做到人均住房面积8平方米以下低收入家庭应保尽保；九是加快实施农村贫困户、残疾人安居工程，改造危房800套；十是改善基层组织办公条件，为每个村（社区）补助办公经费5000元，采取以奖代补推进村委会危房改造，给全市2600多个村民小组赠订报纸。

　　10、加快移民扶贫开发，改善移民生活条件。移民问题事关我市和谐稳定大局，事关全面建设小康社会。我市有移民人口15.45万人，已占全市农村人口的四分之一，收入不足当地农民的三分之一。要高度重视移民扶贫开发，积极争取扶持政策，争取将九甸峡移民列入省上重点扶贫范围，将贫困移民全部纳入农村低保对象。加快移民区基础设施建设，加大生态保护力度。健全移民乡镇公共服务体系，实施移民教育倾斜政策，改善移民乡镇办学条件，提高义务教育普及率，引导移民子女进城就读，资助移民子女接受高等教育。进一步完善领导包抓、对口帮扶制度，动员社会各界关注移民、扶持移民。认真落实移民开发扶持政策，鼓励移民发展设施养殖和特色种植，参加技能培训，外出经商务工，力争移民人均纯收入达到1936元，增长17%。

　　各位代表，今年改革发展稳定的任务异常艰巨，面临的困难和不确定因素较多，我们一定要以科学发展观为指导，推进政府自身建设，强化政府服务职能，提高应对危机、谋划发展的能力和水平，努力实现保增长、扩内需、增效益、促就业的任务目标。坚持民主决策，完善重大事项专家咨询、公开听证和集体决策制度，提高政府决策水平。自觉接受人大法律监督和政协民主监督，高效办理人大代表意见建议和政协提案。坚持依法行政，认真落实依法行政责任制，规范政府议事规则和办事程序，做好规范性文件前置审查，追究执法过错责任。深入开展廉政建设和反腐败斗争，强化审计监督，充分发挥审计的"免疫系统"功能，规范土地出让、工程承包、政府采购、产权交易等招投标程序，加强源头治理，确保权力在阳光下运行。

　　各位代表，回顾过去，我们深感成绩来之不易，展望未来，我们对酒泉的发展充满信心。让我们在中共酒泉市委的正确领导下，团结和带领全市各族人民，克服困难，应对挑战，锐意进取，真抓实干，为完成市二届人大五次会议确定的任务目标而努力奋斗！
